# Large retroperitoneal hematoma following vaginal delivery: a case report

**DOI:** 10.1186/s13256-021-02870-x

**Published:** 2021-05-24

**Authors:** Raha Maroyi, Nyakio Ngeleza, Kiminyi Kalunga, Cikwanine Buhendwa, Usama Shahid, Roland Boij, Denis Mukwege

**Affiliations:** 1 Panzi General Referral Hospital, Bukavu, Democratic Republic of Congo; 2 Université Evangélique en Afrique (U.E.A), Bukavu, Democratic Republic of Congo; 3 The International Center for Advanced Research and Training (ICART), Bukavu, Democratic Republic of Congo; 4 Royal Australian College of Obstetricians and Gynaecologists trainee, James Cook University, Townsville, Australia; 5 The University of Gothenburg, Gothenburg, Sweden

**Keywords:** Retroperitoneal hematoma, Spontaneous vaginal delivery, Delayed diagnosis, Postpartum, Low-income countries

## Abstract

**Background:**

Retroperitoneal hematoma after vaginal delivery is rare but can lead to maternal morbidity and mortality. Diagnosis of this condition is challenging due to its complexity and its nonspecific signs and symptoms. To date, studies and case reports regarding retroperitoneal hematoma are few, particularly in low-income countries where risk factors for this condition may be more prevalent and the prognosis poorer.

**Case presentation:**

We report the case of a 37-year-old multiparous african (Congolese) woman who presented to the emergency department of a large referral hospital in Bukavu, Democratic Republic of the Congo (DRC), 2 weeks after a spontaneous nontraumatic vaginal delivery. She had abdominal pain that began immediately after delivery and progressed throughout the postpartum period. The patient had anemia, hypotension, tachycardia, and a left costo-lumbar arch distorting the body shape on a soft and depressed abdomen. She had visited a private clinic on days 3 and 7 postpartum; however, signs and symptoms of retroperitoneal hematoma went unrecognized. Using abdominal ultrasound, we diagnosed an extensive hematoma in the retroperitoneal space from the left iliac fossa to the left flank. Laparotomy was performed to evacuate the hematoma, and the patient recovered.

**Conclusion:**

Retroperitoneal hematoma following a nontraumatic vaginal delivery is an unusual situation in general obstetrical practice. The knowledge of this potentially life-threatening condition in resource-limited settings enables effective diagnosis and management by ultrasound and laparotomy.

## Background

Retroperitoneal hematoma is a rare and potentially life-threatening complication of pregnancy and childbirth, characterized by acute or subacute bleeding from the abdominal tissues into the retroperitoneum [1, 2]. The lack of specific signs and symptoms associated with retroperitoneal hematoma complicates its diagnosis, and if the condition is not treated promptly, it may endanger the patient's life [1, 3, 4]. However, retroperitoneal hematoma is poorly documented in the literature [1, 2, 4], particularly in low-income countries, where risk factors for this condition may be more prevalent and the prognosis poorer. We present a case of retroperitoneal hematoma that developed progressively during the 14 days following an uncomplicated and nontraumatic vaginal delivery in a resource-limited setting.

## Case presentation

In March 2020, a 37 year-old african (Congolese) woman presented to the emergency department of Panzi General Referral Hospital in Bukavu, Democratic Republic of the Congo (DRC), at 14 days postpartum with generalized cutaneo-mucosal pallor, severe left lateral-hypogastric pain radiating into the homolateral flank, and anemic syndrome. Aside from recent childbirth, she had no specific medical or surgical history and had negative human immunodeficiency virus (HIV) serology. Her medical records indicated that 14 days earlier she had presented to a private clinic in the active phase of labor, at 6 cm of dilatation. Three hours later, she had a normal cephalic delivery of a male newborn weighing 3300 g, with the umbilical cord wrapped once around the neck. No obstetrical maneuver was used to assist the delivery. Her history indicated eight prior vaginal deliveries with no delivery hemorrhage or complications.

On day 3 postpartum, she presented to the private clinic where she had delivered, and reported progressively increasing pain in the left hypochondrium, radiating to the lower limbs and leading to functional impotence of the homolateral lower limb. On day 7 postpartum, she was admitted to the private clinic where she had delivered, with complaint of persistent abdominal pain, and was given a transfusion of one unit of whole blood. On day 8, her signs and symptoms subsided, and she was discharged. Medical records from the clinic where she delivered indicate partially subsided internal bleeding or retroperitoneal hematoma.

### Clinical findings and diagnostic investigations

On day 14 after delivery, the women presented to the emergency department of Panzi Hospital, following a referral from the private clinic where she delivered. On clinical examination, the patient was hypotensive (blood pressure 90/80 mmHg), tachycardic (140 beats per minute), and thin (body mass index 19). The patient reported normal bladder and bowel habits. Medical records indicated that 4 weeks before her delivery, she had normal hemoglobin (12.7 g/dl) but severe leukocytosis (161 x10^9^/l). At the time of the clinical examination at Panzi Hospital, her hemoglobin had dropped to 6.5 g/dl, her hematocrit level was 16.0%, leukocytosis had progressed to 172 x10^9^/l, and thrombocytosis was present (platelets 468 x 10^9^ /l); the clotting profile was normal.

**Fig. 1:**
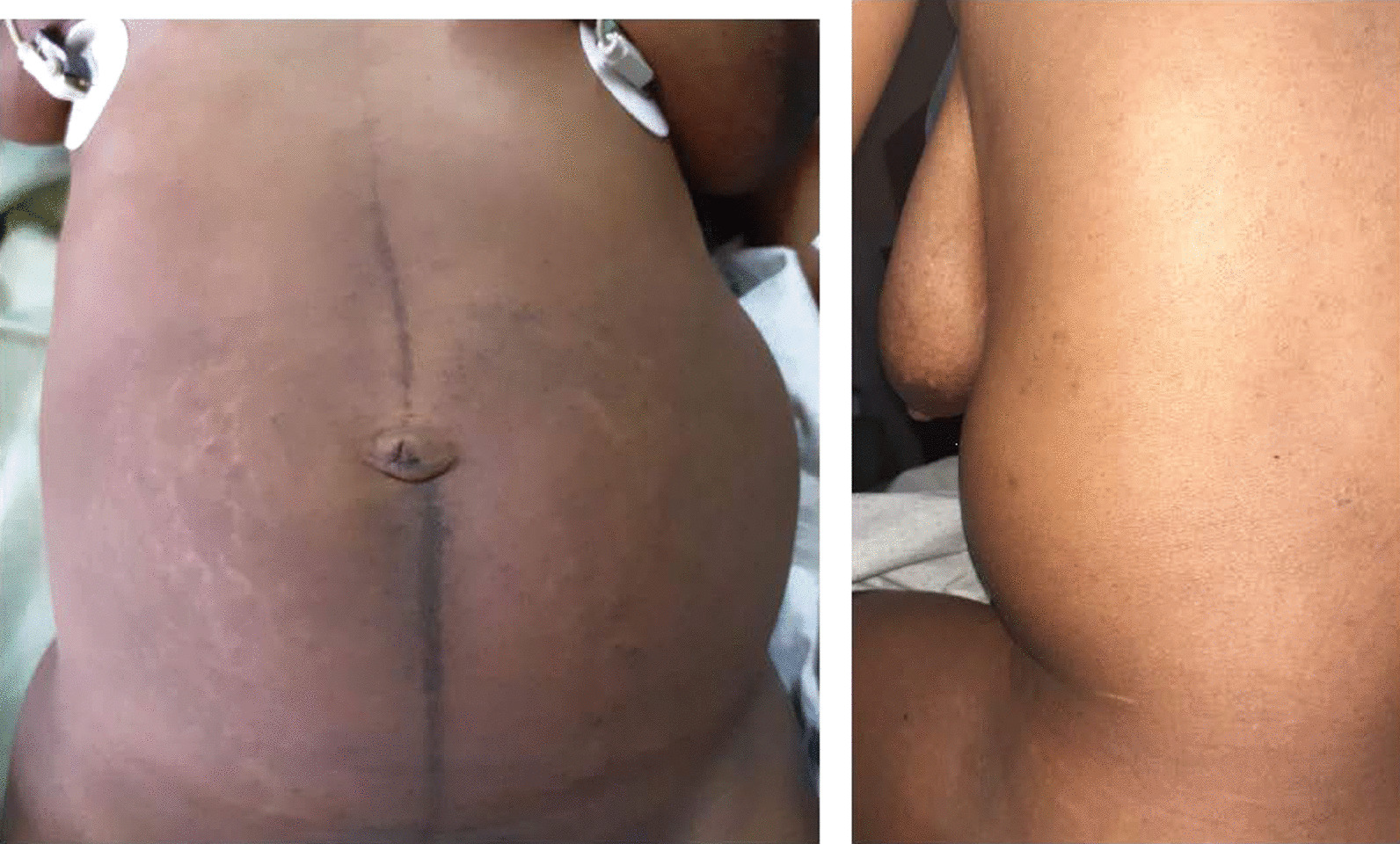
Left costo-lumbar arch distorting the body shape

On pelvic examination, we found no signs of vulvar or vaginal hematoma, the cervix was atraumatic, and the uterus was in the normal pelvic position without pain on mobilization; the cul-de-sac was not bulging. Her abdomen was soft and depressed, with a left costo-lumbar arch distorting the body shape (Fig. 1). We noted deep abdominal tenderness in the left hemi-abdomen and the uterus in the pelvis that was suggestive of a hematoma.

**Fig. 2:**
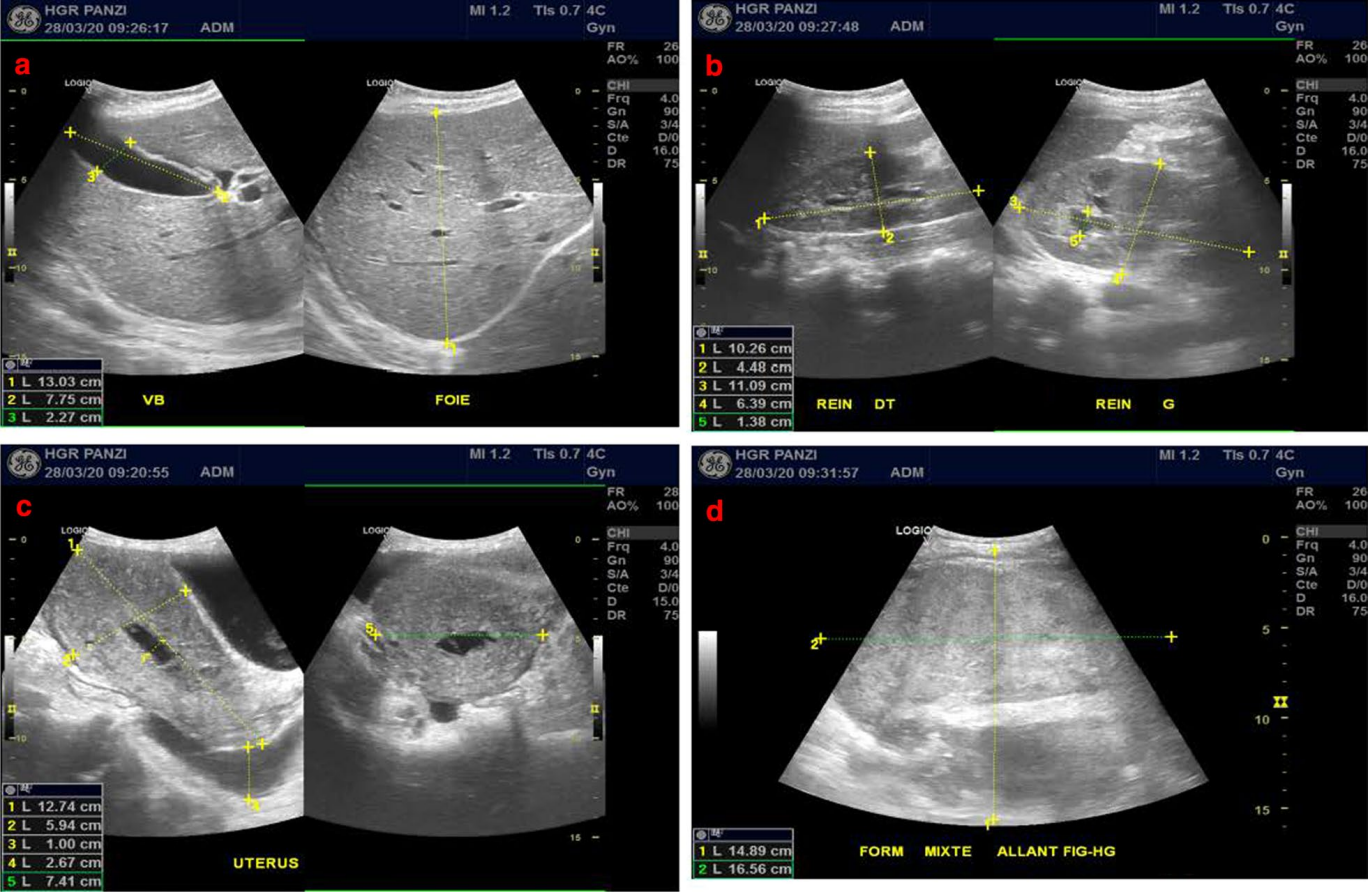
Ultrasound images showing the liver (**a**), kidneys (**b**), uterus and pelvis (**c**), hematoma (**d**)

Abdominopelvic ultrasonographic findings revealed a mixed hyperechoic formation measuring 148.9 x 59.4 mm that extended from the left iliac fossa to the left flank; both kidneys were normal. The uterus was normal (127.4 x 59.4 mm), and there was free fluid in the pouch of Douglas of 26.7 mm (Fig. 2).

### Management

**Fig. 3:**
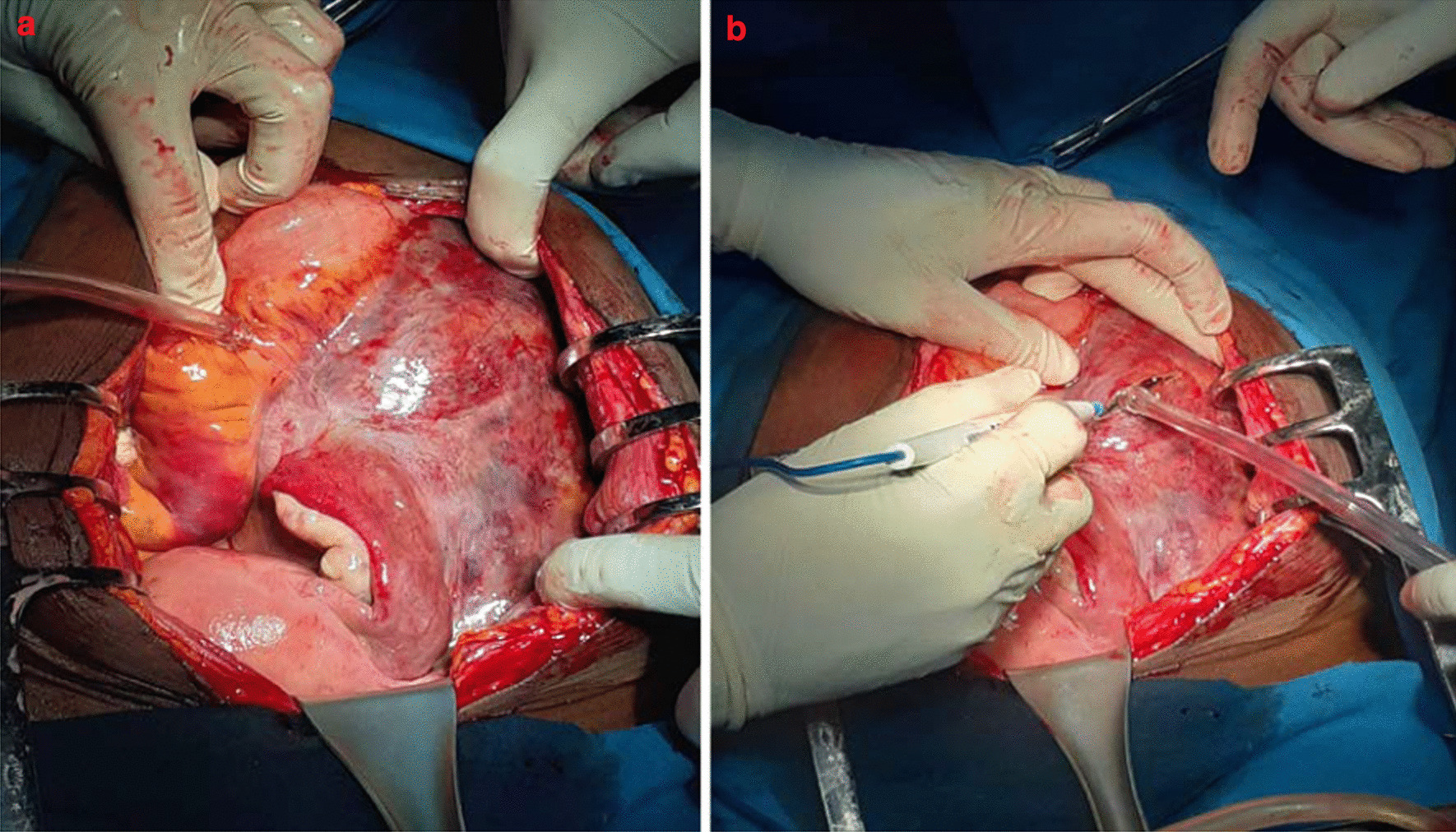
**a** Retroperitoneal hematoma, **b** peritoneal incision

In view of severe anemia with tachycardia, the patient was immediately given a transfusion of four units of whole blood. An exploratory laparotomy was performed with concomitant intravenous administration of antibiotic therapy and 1 g of tranexamic acid. The laparotomy revealed a large retroperitoneal hematoma (Fig. 3), which was evacuated by making a 3 cm median retroperitoneal incision from the uterine horn into the lumbar fossa, followed by evacuation of 3000 ml of clots and blood. The retroperitoneum was left unsutured, and a lamellar abdominal drain was inserted. Upon completing the evacuation, there was no active bleeding.

### Outcome

On day 15, (1 day following laparotomy with evacuation of the hematoma), the patient was stable with normal blood pressure (106/65 mmHg), a normal pulse rate (85 beats/minute), and a normal respiration rate (20 breaths per minute). The daily volume of serous fluid collected in the 24 hours following laparotomy was small (approximately 600 ml), indicating that the hematoma had been stabilized. The patient reported a significant decrease in pain; physiological and ultrasound findings indicated a good recovery. The patient was discharged without complications on day 22 (7 days following laparotomy).

## Discussion

Retroperitoneal hematoma is a rare complication of normal pregnancy and childbirth, with the most common pregnancy-related hematomas occurring in the vulva and the vagina [1, 5]. Pregnancy-associated retroperitoneal hematoma is distinguished by its timing of onset, which can occur during pregnancy or up to 4 weeks postpartum. Onset can be acute or subacute [1, 4, 5] and may result from rupture of an arterial aneurysm, or injury due to obstetric trauma to the pelvic venous plexus, which is dilated and fragile during pregnancy [4, 5]. Several risk factors for retroperitoneal hematoma have been identified: coagulation disorders, congenital Ehlers-Danlos syndrome type 4 with rupture of retroperitoneal vessels, prior use of anticoagulant treatment, severe preeclampsia, HELLP [hemolysis, elevated liver enzymes, low platelet count] syndrome, multiparity, macrosomia, multiple pregnancy, prolonged active phase of labor, traumatic delivery, and manual delivery of the placenta [1, 2, 6]. Several of these factors are prevalent in resource-poor settings, including multiparity and multiple pregnancy.

Following a vaginal delivery, diagnosis of retroperitoneal hematoma is usually made in the immediate or late postpartum period due to either persistent pain or hemorrhagic shock. Ultrasound, while not as accurate as computed tomography (CT) scan or magnetic resonance imaging (MRI), is an acceptable alternative method of diagnosing retroperitoneal hematoma [2, 4, 9]. An abdominopelvic MRI or CT scan of the site of pain will determine the source of bleeding and hematoma location and diameter [1, 6]. Early diagnosis and management of retroperitoneal hematoma is essential to preventing serious and life-threatening consequences [1–5]; however, diagnosis is frequently delayed due to difficulties in recognizing the clinical signs and symptoms. Bleeding associated with retroperitoneal hematoma is frequently only internal, and symptoms are often nonspecific [3, 5, 7]. Often there is a single hematoma formation without associated visible bleeding, and abdominal pain can mimic a thrombus. Sometimes pain at onset can give the impression of a benign condition [6].

Management of retroperitoneal hematoma may be conservative or surgical and will depend on the size and evolution of the hematoma, as well as the hemodynamic status of the patient [1, 6]. A conservative approach is recommended for patients who are hemodynamically stable after receipt of a blood transfusion, and in whom the hematoma is not expanding and symptoms of pressure are absent [1, 4]. In clinically stable patients, the retroperitoneal space may restrict expansion of the hematoma following spontaneous self-stamping [5], resulting in an inactive retroperitoneal hematoma, which frequently undergo spontaneous reabsorption [4, 5]. In patients who are hemodynamically unstable but do not have an extensive hematoma, vascular embolization has been shown to have good outcomes [1, 10]. However, vascular embolization requires close coordination of clinical specialists, including an interventional radiologist, gynecologist, and anesthetist [6], and is not feasible in many resource-limited settings [1–3, 10]. Finally, laparotomy is indicated as a last resort in clinically unstable patients who have an extensive hematoma, due to risk of hemorrhagic shock, disseminated intravascular coagulation (DIC), and sepsis [1, 2].

Our case report demonstrates that in resource-limited settings, retroperitoneal hematoma in postpartum woman can be effectively managed in hospitals where abdominal surgery is performed, and CT scan, or at least ultrasound equipment, is available. Specifically, in a women presenting to the emergency department with severe anemia and hemodynamic instability 2 weeks postpartum, we made an accurate diagnosis guided only by ultrasound, and subsequently successfully evacuated a large hematoma. Our case study highlights the complexity of diagnosing retroperitoneal hematoma, which in this case manifested with nonspecific signs and symptoms that progressed during the 2 weeks following delivery, and culminated in severe anemia that was not appropriately diagnosed at the private clinic where the patient first presented.

While diagnosis of retroperitoneal hematoma is challenging in any setting, it is particularly so in resource-limited settings, where relevant clinical experience and medical equipment may be lacking and pregnant women are frequently discharged shortly after delivery, without adequate follow-up. Thus, diagnosis of retroperitoneal hematoma may be delayed and result in a poorer prognosis in low-income countries compared to industrialized countries. While few cases of retroperitoneal hematoma are reported from low-income countries, we speculate that this may be due to low recognition of this condition in routine clinical practice.

## Conclusion

Our case report emphasizes the lack of awareness of postpartum retroperitoneal hematoma in general obstetric practice. Patients can develop symptoms of pelvic hematoma immediately after delivery or may take several days, as in the present case. Hence, differential diagnosis of pelvic hematoma should be considered in patients with persistent abdominal pain with progressively developing anemia without any evidence of external bleeding or postpartum hemorrhage.

In a resource-limited setting, ultrasound and evacuation laparotomy can effectively diagnose and treat retroperitoneal hematoma, even when the hematoma is large and unstable.

## Data Availability

All data generated and analyzed in this study are included in the manuscript.
